# Emergence of Hypervirulent Carbapenem-Resistant Klebsiella pneumoniae Coharboring a *bla*_NDM-1_-Carrying Virulent Plasmid and a *bla*_KPC-2_-Carrying Plasmid in an Egyptian Hospital

**DOI:** 10.1128/mSphere.00088-21

**Published:** 2021-05-19

**Authors:** Mohamed Abd El-Gawad El-Sayed Ahmed, Yanxian Yang, Yongqiang Yang, Bin Yan, Guanping Chen, Reem Mostafa Hassan, Lan-Lan Zhong, Yuan Chen, Adam P. Roberts, Yiping Wu, Ruowen He, Xiaoxue Liang, Mingyang Qin, Min Dai, Liyan Zhang, Hongyu Li, Fan Yang, Lingqing Xu, Guo-Bao Tian

**Affiliations:** a Department of Microbiology, School of Basic Medical Science, Xinxiang Medical University, Xinxiang, China; b Department of Microbiology, Zhongshan School of Medicine, Sun Yat-sen University, Guangzhou, China; c Key Laboratory of Tropical Diseases Control (Sun Yat-sen University), Ministry of Education, Guangzhou, China; d Department of Microbiology and Immunology, Faculty of Pharmaceutical Sciences and Drug Manufacturing, Misr University for Science and Technology, 6th of October City, Egypt; e School of Pharmaceutical Sciences (Shenzhen), Sun Yat-sen University, Guangzhou, China; f Department of Neonatal Surgery, Guangzhou Women and Children's Medical Center, Guangzhou, China; g Sun Yat-Sen University School of Medicine, Guangzhou, China; h Department of Clinical and Chemical Pathology, Faculty of Medicine, Cairo University, Cairo, Egypt; i Department of Tropical Disease Biology, Liverpool School of Tropical Medicine, Liverpool, United Kingdom; j Centre for Drugs and Diagnostics, Liverpool School of Tropical Medicine, Liverpool, United Kingdom; k School of Laboratory Medicine, Chengdu Medical College, Chengdu, China; l Department of Clinical Laboratory, Guangdong Provincial People’s Hospital/Guangdong Academy of Medical Sciences, Guangzhou, Guangdong, China; m Department of Clinical Laboratory, Sun Yat-sen Memorial Hospital, Sun Yat-sen University, Guangzhou, China; n Department of Clinical Laboratory, The Sixth Affiliated Hospital of Guangzhou Medical University, Qingyuan People's Hospital, Qingyuan, China; o School of Medicine, Xizang Minzu University, Xianyang, Shaanxi, China; Antimicrobial Development Specialists, LLC

**Keywords:** *Klebsiella pneumoniae*, NDM-1, KPC-2, hybrid plasmid, virulent plasmid, Egypt

## Abstract

The emergence of carbapenem-resistant Klebsiella pneumoniae (CRKP) isolates in Egyptian hospitals has been reported. However, the genetic basis and analysis of the plasmids associated with carbapenem-resistant hypervirulent K. pneumoniae (CR-HvKP) in Egypt have not been presented. Therefore, we attempted to decipher the plasmid sequences that are responsible for transferring the determinants of carbapenem resistance, particularly *bla*_NDM-1_ and *bla*_KPC-2_. Out of 34 K. pneumoniae isolates collected from two tertiary hospitals in Egypt, 31 were CRKP. Whole-genome sequencing revealed that our isolates were related to 13 different sequence types (STs). The most prevalent ST was ST101, followed by ST383 and ST11. Among the CRKP isolates, one isolate named EBSI036 has been reassessed by Nanopore sequencing. Genetic environment analysis showed that EBSI036 carried 20 antibiotic resistance genes and was identified as a CR-HvKP strain: it harbored four plasmids, namely, pEBSI036-1-NDM-VIR, pEBSI036-2-KPC, pEBSI036-3, and pEBSI036-4. The two carbapenemase genes *bla*_NDM-1_ and *bla*_KPC-2_ were located on plasmids pEBSI036-1-NDM-VIR and pEBSI036-2-KPC, respectively. The IncFIB:IncHI1B hybrid plasmid pEBSI036-1-NDM-VIR also carried some virulence factors, including the regulator of the mucoid phenotype (*rmpA*), the regulator of mucoid phenotype 2 (*rmpA2*), and aerobactin (*iucABCD* and *iutA*). Thus, we set out in this study to analyze in depth the genetic basis of the pEBSI036-1-NDM-VIR and pEBSI036-2-KPC plasmids. We report a high-risk clone ST11 KL47 serotype of a CR-HvKP strain isolated from the blood of a 60-year-old hospitalized female patient from the intensive care unit (ICU) in a tertiary care hospital in Egypt, which showed the cohabitation of a novel hybrid plasmid coharboring the *bla*_NDM-1_ and virulence genes and a *bla*_KPC-2_-carrying plasmid.

**IMPORTANCE** CRKP has been registered in the critical priority tier by the World Health Organization and has become a significant menace to public health. The emergence of CR-HvKP is of great concern in terms of both disease and treatment. In-depth analysis of the carbapenemase-encoding and virulence plasmids may provide insight into ongoing recombination and evolution of virulence and multidrug resistance in K. pneumoniae. Thus, this study serves to alert contagious disease clinicians to the presence of hypervirulence in CRKP isolates in Egyptian hospitals.

## OBSERVATION

Several studies have reported the emergence of carbapenem-resistant Klebsiella pneumoniae (CRKP) isolates in Egyptian hospitals (1–4); however, to the best of our knowledge, the genetic basis and analysis of the plasmids associated with CR-hypervirulent K. pneumoniae (CR-HvKP) in Egypt have not been presented. Furthermore, carbapenem resistance has been reported to be associated with increased length of hospital stay and mortality of bloodstream infection (BSI) patients in low- and middle-income countries (5). Therefore, we sought to analyze in depth the genetic basis of pEBSI036-1-NDM-VIR (a novel hybrid plasmid harboring *bla*_NDM-1_ and virulence genes) and pEBSI036-2-KPC (a *bla*_KPC-2_-carrying plasmid), which have been identified in a clinical K. pneumoniae strain from a blood sample of a patient in Egypt.

A total of 34 nonduplicate K. pneumoniae isolates were recovered from the blood of hospitalized patients in two tertiary care hospitals, namely, El-Demerdash Hospital (Cairo, Egypt) and the National Cancer Institute (Cairo, Egypt), in the period between June 2017 and March 2018 as a part of a study for the monitoring of antimicrobial resistance. Our isolates were selected based on their clinical characteristics, where all of them were primarily identified by Vitek 2 and matrix-assisted laser desorption ionization–time of flight mass spectrometry (MALDI-TOF MS) as K. pneumoniae causing bloodstream infections (BSIs), among which 31 were confirmed phenotypically and genotypically as CRKP isolates. Overall, the 34 isolates were isolated from the blood of 55.9% (19/34) female and 44.1% (15/34) male hospitalized patients from 9 days to 75 years of age. MICs of all 34 isolates were determined for 17 antibiotics using the agar microdilution method according to CLSI (6), except for tigecycline and colistin, for which MICs were obtained by the broth microdilution method according to EUCAST (7). Out of 34 isolates, 91.2% (31/34) were resistant to ertapenem, while 73.5% (25/34) and 61.8% (21/34) were resistant to imipenem and meropenem, respectively. However, all isolates were susceptible to colistin.

All isolates were assessed by whole-genome sequencing (WGS) using an Illumina HiSeq 2000 platform. *In silico* multilocus sequence typing showed that our isolates belong to 13 different sequence types (STs). The most prevalent ST was ST101 (13/34 [38.2%]), followed by ST383 (5/34 [14.7%]). One isolate, EBSI036, belongs to ST11: ST11 is the dominant ST clone responsible for the prevalence of CRKP worldwide and is considered an emerging high-risk clone (1, 8–10). Among 31 CRKP isolates, the prevalence of carbapenemase genes *bla*_NDM-1_ and *bla*_OXA-48_ was 45.2% (14/31)—in addition, four strains carried both genes. Moreover, strain EBSI036 coharbors *bla*_NDM-1_ and *bla*_KPC-2_. According to the clinical data, the K. pneumoniae strain EBSI036 was isolated from the blood of a 60-year-old female patient 2 days after admission to the Gastroenterology Department of El-Demerdash Hospital with symptoms of pneumonia, diarrhea, and fever. The patient’s symptoms improved following the administration of intravenous ceftriaxone and colistin, and she was discharged from the hospital 8 days posthospitalization. With the further genome analysis of EBSI036, the plasmid-associated virulence determinants *rmpA*/*rmpA2*, *iucABCD*, and *iutA* in this strain were predicted using the Virulence Factor Database (VFDB [http://www.mgc.ac.cn/VFs/main.htm]). EBSI036 was determined as a KL47 capsular serotype by using Kaptive software (https://github.com/katholt/Kaptive). The serotype KL47 was the most reported type among CRKP infections in Asia (11–13). The virulence level of EBSI036 was confirmed using the Galleria mellonella larva model as previously described (11, 14) (see Fig. S1 in the supplemental material). These results revealed that EBSI036 is a CR-HvKP strain.

10.1128/mSphere.00088-21.1 FIG S1 Virulence potential of K. pneumoniae strain EBSI036 as depicted in a Galleria mellonella infection model with an inoculum of 1 × 10^4^ CFU. Download FIG S1, TIF file, 0.3 MB. Copyright © 2021 Ahmed et al. 2021 Ahmed et al. https://creativecommons.org/licenses/by/4.0/ This content is distributed under the terms of the Creative Commons Attribution 4.0 International license.

As EBSI036 coharbors two carbapenem genes besides the plasmid-mediated virulence genes, we have further analyzed the characteristics of the related fully sequenced plasmids by using a long-read MinION sequencer (Oxford Nanopore Technologies, Oxford, United Kingdom). Genomic analysis showed that EBSI036 included a 5,513,124-bp chromosome and four plasmids, namely, pEBSI036-1-NDM-VIR (347,365 bp), pEBSI036-2-KPC (129,869 bp), pEBSI036-3 (10,060 bp), and pEBSI036-4 (5,596 bp) (see Table S1 in the supplemental material). Twenty antimicrobial resistance genes, including six β-lactamase-encoding genes, were identified in EBSI036 by using ABRicate version 0.5 (https://github.com/tseemann/abricate) and aligning genome sequences to the ResFinder database. Of these, *bla*_SHV-11_, *oqxB*, *oqxA*, and *fosA6*, were identified in the EBSI036 chromosome. The two carbapenemase genes *bla*_NDM-1_ and *bla*_KPC-2_ were located on plasmids pEBSI036-1-NDM-VIR and pEBSI036-2-KPC, respectively. In addition, 86 putative virulence genes were annotated in the genome of EBSI036, including genes coding for fimbriae, capsule, yersiniabactin, iron-enterobactin, mucoid, and aerobactin (see Table S2 in the supplemental material).

10.1128/mSphere.00088-21.3 TABLE S1 Overall features of the genome and minimum inhibitory concentrations (MICs) for K. pneumoniae EBSI036. Download Table S1, DOCX file, 0.03 MB. Copyright © 2021 Ahmed et al. 2021 Ahmed et al. https://creativecommons.org/licenses/by/4.0/ This content is distributed under the terms of the Creative Commons Attribution 4.0 International license.

10.1128/mSphere.00088-21.4 TABLE S2 Putative virulence genes detected on the K. pneumoniae EBSI036 chromosome. Download Table S2, XLS file, 0.04 MB. Copyright © 2021 Ahmed et al. 2021 Ahmed et al. https://creativecommons.org/licenses/by/4.0/ This content is distributed under the terms of the Creative Commons Attribution 4.0 International license.

**FIG 1:**
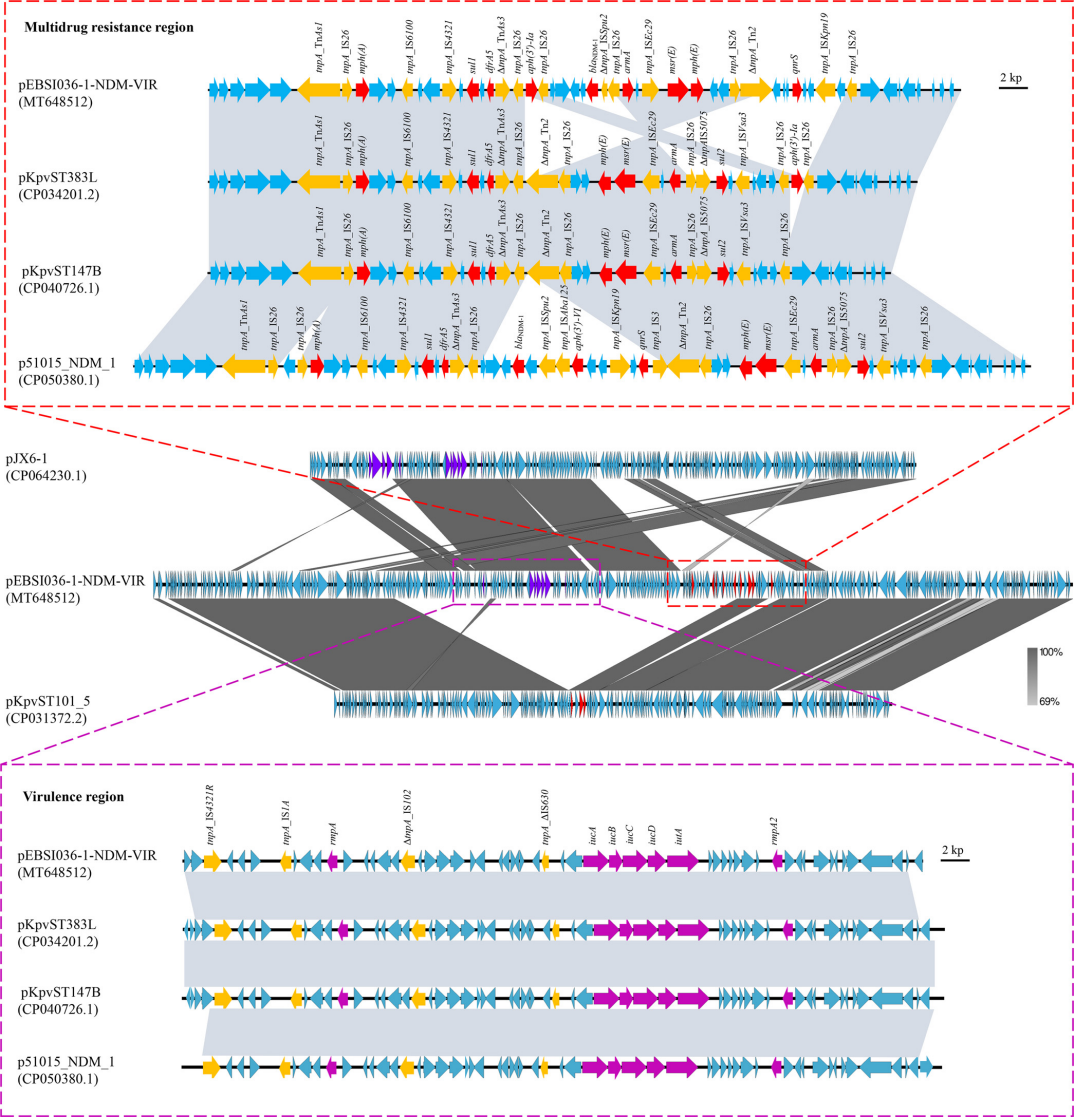
Structure analysis of pEBSI036-1-NDM-VIR. Major structural features of plasmid pEBSI036-1-NDM-VIR were compared with those of plasmids pKpvST101_5 (GenBank accession no. CP031372.2) and pJX6-1 (GenBank accession no. CP064230.1). The comparative schematic diagrams of resistance regions and virulence regions in plasmids pEBSI036-1-NDM-VIR, pKpvST383L (GenBank accession no. CP034201.2), pKpvST147B_virulence (GenBank accession no. CP040726.1), and p51015_NDM_1 (GenBank accession no. CP050380.1) are shown. Gray shading indicates shared regions with a high degree of homology. Red and purple represent the antibiotic resistance and virulence genes, respectively, and yellow represents the insertion sequences and transposons.

Hybrid plasmids that harbor resistance and virulence genes in a single genetic environment have been reported recently in various K. pneumoniae isolates, including the high risk of virulence clone ST23 and multidrug resistance (MDR) clone ST11 (15–17). Herein, the largest pEBSI036-1-NDM-VIR plasmid belongs to an IncFIB:IncHI1B hybrid plasmid. BLASTn showed that pEBSI036-1-NDM-VIR shared >99% identity with plasmids pKpvST383L (CP034201.2), pKpvST147B_virulence (CP040726.1), and p51015_NDM_1 (CP050380.1), with query coverages of 97 to 99% (see Fig. S2 in the supplemental material). The backbone region of pEBSI036-1-NDM-VIR almost covered the complete sequence of the MDR plasmid pKpvST101_5, with a length of 210,661 bp (CP031372.2) (Fig. 1). Most of the remaining sequences (∼130 kb) of pEBSI036-1-NDM-VIR were similar to those of the virulence plasmid pJX6-1, with a length of 228,974 bp (CP064230.1) (Fig. 1).

10.1128/mSphere.00088-21.2 FIG S2 Sequence alignment analysis among plasmids pEBSI036-1-NDM-VIR and pKpvST383L (GenBank accession no. CP034201.2), pKpvST147B_virulence (GenBank accession no. CP040726.1), and p51015_NDM_1 (GenBank accession no. CP050380.1). Download FIG S2, TIF file, 2.3 MB. Copyright © 2021 Ahmed et al. 2021 Ahmed et al. https://creativecommons.org/licenses/by/4.0/ This content is distributed under the terms of the Creative Commons Attribution 4.0 International license.

An ∼38-kb MDR region in pEBSI036-1-NDM-VIR harbored carbapenemase-encoding gene *bla*_NDM-1_ and another eight resistance genes: *mph*(A), *sul1*, *dfrA5*, *aph*(3′)*-Ia*, *armA*, *msr*(E), *mph*(E), and *qnrS*. A truncated transposon, ΔTn*As1* (Tn*3* family [6,694 bp]), and IS*26* elements (IS*6* family [820 bp]) were located upstream of *mph*(A). The *mph*(A) gene and the downstream complete IS*6100* sequence (family IS*6* [880 bp]) were separated by two open reading frames (ORFs). *sul1* and *dfrA5* were surrounded by IS*4321* (family IS*110* [1,327 bp]), ΔTn*As3* (Tn*3* family [18,375 bp]), and IS*26* elements. This fragment with 15,448 bp containing the resistance genes mentioned above was similar to plasmid pKpvST383L (Fig. 1). The *aph*(3′)*-Ia* gene was flanked by IS*26* elements; a similar structure was also found downstream of the resistance region in pKpvST383L. The segment IS*26*-*armA*-IS*26*-*msr*(E)-*mph*(E)-ORF-ORF-IS*26*-ΔTn*2* in pEBSI036-1-NDM-VIR was also found to be identical to an inverted sequence in pKpvST383L. In addition, the *bla*_NDM-1_ and *qnrS* genes were on either side of this fragment, while they were absent in pKpvST383L. By comparing the complete sequences of pEBSI036-1-NDM-VIR and pKpvST383L, it was found that pKpvST383L had another resistance region (26,683 bp) carrying *bla*_NDM-5_ and *bla*_OXA-9_. Compared with plasmid p51015_NDM_1, the resistance region of plasmid pEBSI036-1-NDM-VIR lacked the *aph*(3′)*-VI* and *sul2* genes (Fig. 1). In pEBSI036-1-NDM-VIR, the MDR region contained six IS*26* elements and other transposon elements. Some studies demonstrated that the resistance loci containing IS*26* can be hot spots for the capture of further resistance genes to constitute a novel MDR region (18).

A set of virulence genes was detected in pEBSI036-1-NDM-VIR with increased colonization and infection-producing capabilities, including *rmpA* and *rmpA2* for the hypermucoviscous phenotype and *iucABCD* and *iutA*, associated with virulence (19). The ∼39-kb region harboring virulence genes exhibited high similarity (99.9% identity and 98% query coverage) to pKpvST383L, pKpvST147B_virulence, and p51015_NDM_1 (Fig. 1).

**FIG 2:**
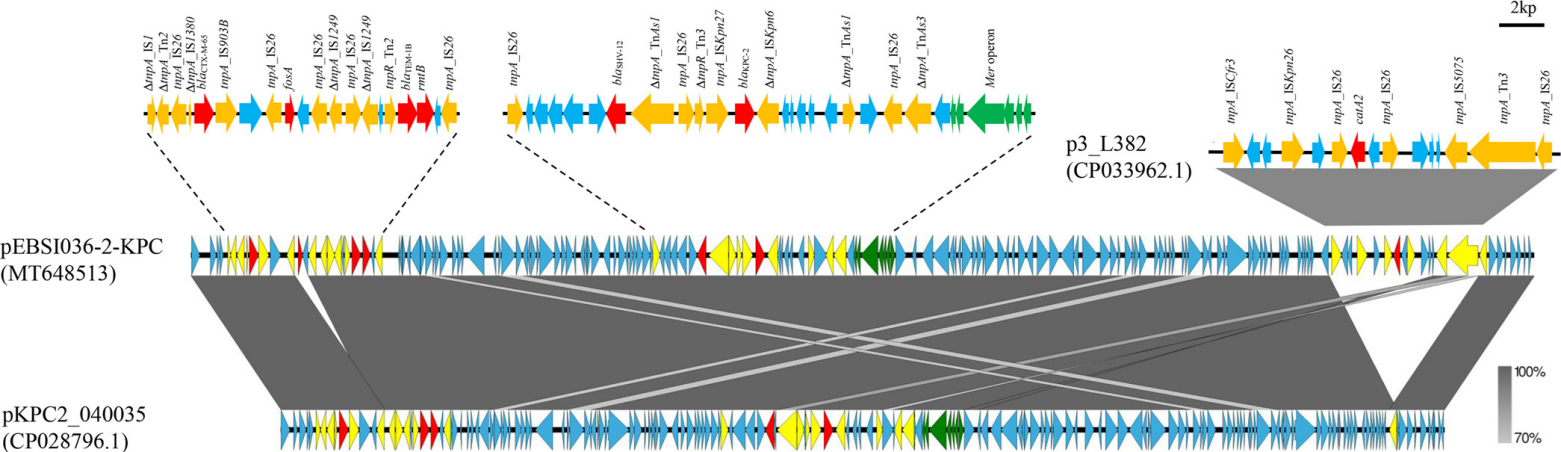
Sequence alignment analysis among plasmids pEBSI036-2-KPC, pKPC2_040035 (GenBank accession no. CP028796.1), and p3_L382 (GenBank accession no. CP033962.1). Red and green represent the antibiotic resistance and heavy metal resistance genes, respectively, and yellow represents the insertion sequences and transposons.

Comparative analysis showed that the IncR:IncFII-type plasmid pEBSI036-2-KPC had 98 to 99% query coverages and 99.9% nucleotide identity to the following plasmids: pKP19-2029-KPC2 (CP047161.1), p69-2 (CP025458.1), and p16HN-263_KPC (CP045264.1). The pEBSI036-2-KPC plasmid carried the carbapenemase-encoding gene *bla*_KPC-2_ and three β-lactamase-encoding genes: *bla*_CTX-M-65_, *bla*_TEM-1B_, and *bla*_SHV-12_. The pEBSI036-2-KPC plasmid had additional resistance genes: *catA2*, *fosA3*, and *rmtB*. These resistance genes were located in two regions (Fig. 2). The *bla*_KPC-2_ and *bla*_SHV-12_ genes were separated by sequence ΔTn*As1*-IS*26*-ΔTn*3*-IS*Kpn27*, and IS*Kpn6* was located downstream of *bla*_KPC-2_. Sequence downstream of *bla*_KPC-2_ contained a *mer* operon responsible for mercuric resistance and transposon elements (ΔTn*As1*-IS*26*-ΔTn*As3*). This segment carrying *bla*_KPC-2_, *bla*_SHV-12_, and a *mer* operon, was highly similar to those of other plasmids, such as pKP1034 (20). There was another MDR region (15,254 bp) that consisted of *bla*_CTX-M-65_, *fosA3*, *bla*_TEM-1B_, and *rmtB* genes and five IS*26* fragments (Fig. 2). The basic structure of pEBSI036-2-KPC was similar to that of plasmid pKPC2_040035 (CP028796.1) (99.98% identity and 88% query coverage), except for two regions. One locus (10,379 to 10,795 bp) carried *fosA3*, which was flanked by IS*26*. The other region contained a ΔIS*Cfr3-*IS*Kpn26*-IS*26*-*catA2*-IS*26*-IS*5075*-ΔTn*3*-IS*26* structure with a length of 15,042 bp, which was the same as plasmid p3_L382 (CP033962.1), with 100% query coverage and 99.99% nucleotide identity (Fig. 2). Both *fosA3* and *catA2* were flanked by IS*26*, as previously reported (20, 21). That evidence emphasizes the role of insertion elements such as IS*26* in regulating insertion and deletion of resistance genes again.

In conclusion, we have reported a high-risk clone of ST11 KL47 of a CR-HvKP strain isolated from the blood of a patient from an ICU in Egypt, which coharbors two plasmids: one is a novel hybrid plasmid harboring the carbapenemase gene *bla*_NDM-1_ and virulence genes, and the other carries *bla*_KPC-2_. Further countrywide surveillance studies are needed to elucidate the rate of prevalence of this high-risk clone in Egypt and its burden on hospital-acquired infections.

### Accession numbers.

The sequences of the plasmids pEBSI036-1-NDM-VIR and pEBSI036-2-KPC have been deposited in GenBank under accession no. MT648512 and MT648513.
