# Supplementary figures and images for: Emergence of Hypervirulent Carbapenem-Resistant Klebsiella pneumoniae Coharboring a blaNDM-1-Carrying Virulent Plasmid and a blaKPC-2-Carrying Plasmid in an Egyptian Hospital
Source: mSphere. 2021 May 19;6(3):e00088-21. doi: 10.1128/mSphere.00088-21 (PMC8265623; doi:10.1128/mSphere.00088-21)

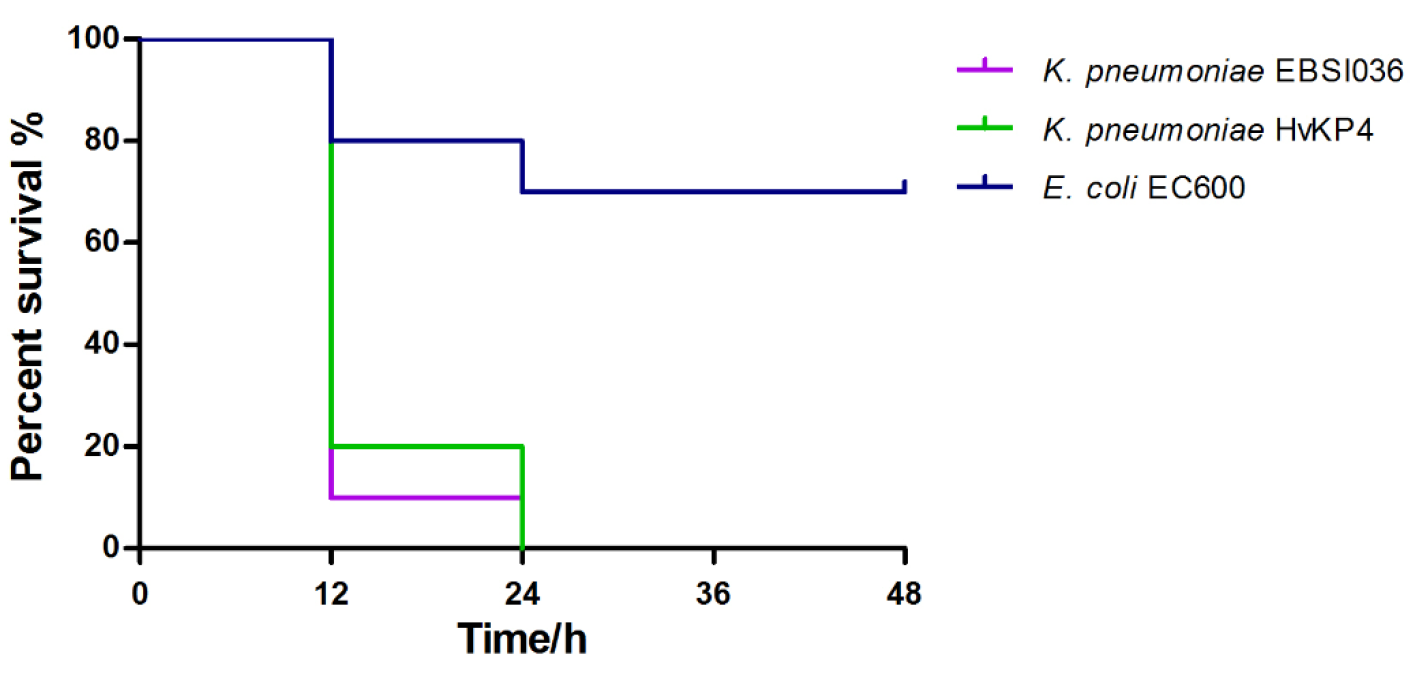

Supplement: FIG S1 [file msphere.00088-21-sf001.tif]

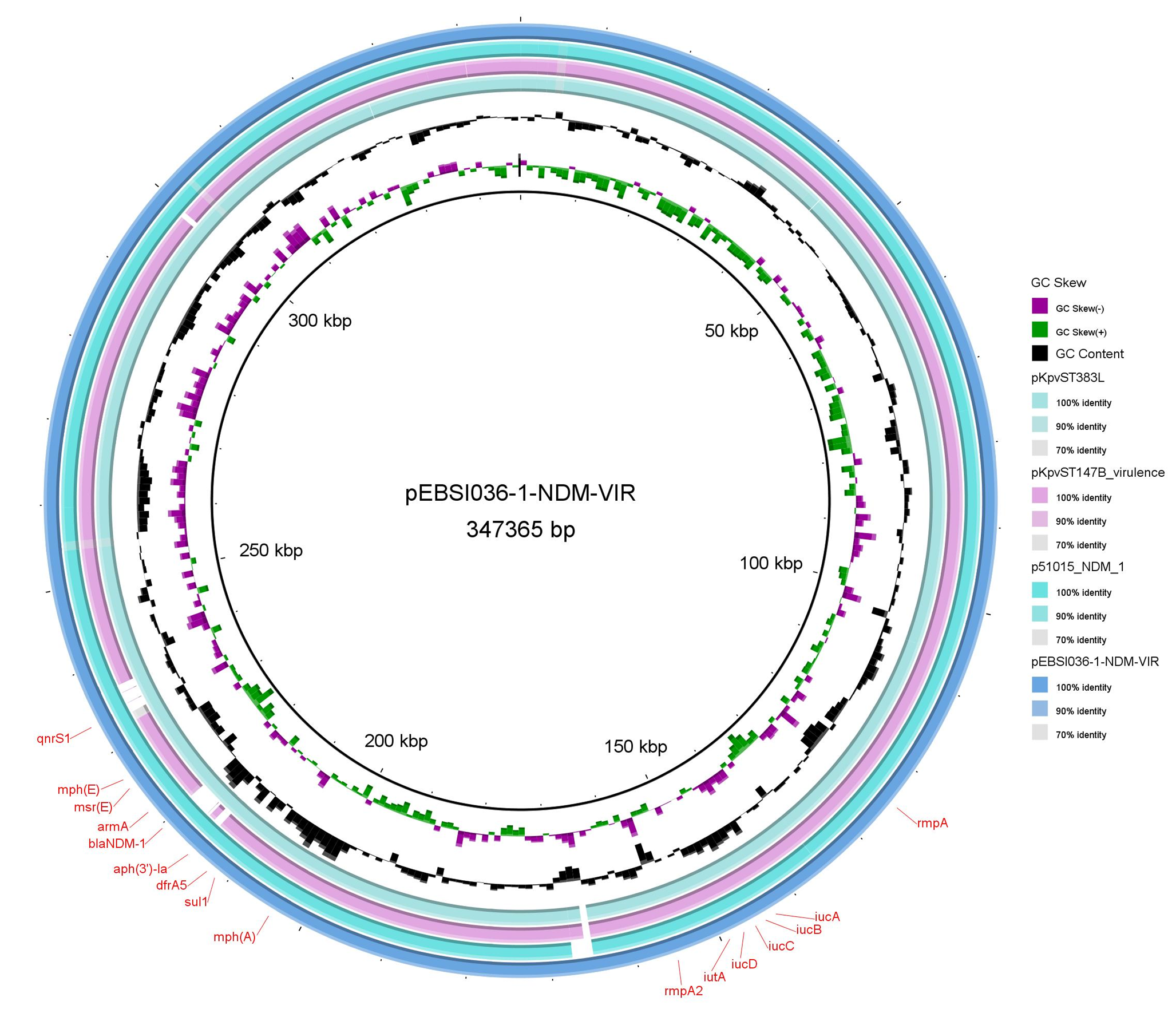

Supplement: FIG S2 [file msphere.00088-21-sf002.tif]
