# Supplementary material for: Emergence of Hypervirulent Carbapenem-Resistant Klebsiella pneumoniae Coharboring a blaNDM-1-Carrying Virulent Plasmid and a blaKPC-2-Carrying Plasmid in an Egyptian Hospital
Source: mSphere. 2021 May 19;6(3):e00088-21. doi: 10.1128/mSphere.00088-21 (PMC8265623; doi:10.1128/mSphere.00088-21)
Supplement: TABLE S1 [file msphere.00088-21-st001.docx]

**Table S1.** Overall features of genome and Minimum Inhibitory Concentrations (MICs) for the *K. pneumoniae* EBSI036

| Parameter | Chromosome | pEBSI036-1-NDM-VIR | pEBSI036-2-KPC | pEBSI036-3 | pEBSI036-4 | MIC s (mg/L) | |
| --- | --- | --- | --- | --- | --- | --- | --- |
| Size (bp) | 5 513 124 | 347 365 | 129 869 | 10 060 | 5 596 | **IMP** | >16 |
| Plasmid type | - | IncFIB:IncHI1B | IncR:IncFII | ColRNAI | ColRNAI | **MEM** | >16 |
| Resistance determinants | *oqxB*  *oqxA*  *bla*_SHV-11_  *fosA6* | *mph(A)*  *sul1*  *dfrA5*  *aph(3')-Ia*  *bla*_NDM-1_  *armA*  *msr(E)*  *mph(E)*  *qnrS1* | *bla*_CTX-M-65_  *fosA3*  *bla*_TEM-1B_  *rmtB*  *bla*_SHV-12_  *bla*_KPC-2_  *catA2* | - | - | **ETP**  **TZP**  **CTX**  **CAZ**  **FEP**  **ATM**  **GEN**  **AMK**  **CIP** | >16  >512  >128  >128  128  >128  >256  >256  64 |
| Virulence genes | 79 putative virulence factor genes were showed in Table S2 | *rmpA*  *rmpA2*  *iucA*  *iucB*  *iucC*  *iucD*  *iutA* | - | - | - | **FOS**  **SXT**  **CHL**  **TET**  **TGC**  **CST** | >512  >16  >256  4  1  0.25 |
| Accession no. | - | MT648512 | MT648513 | - | - |  |  |

IPM, imipenem; MEM, meropenem; ETP, ertapenem; TZP, piperacillin-tazobactam; CTX, cefotaxime; CAZ, ceftazidime; FEP, cefepime; ATM, aztreonam; GEN, gentamicin; AMK, amikacin; CIP, ciprofloxacin; FOS, fosfomycin; SXT, trimethoprim-sulfamethoxazole; CHL, chloramphenicol; TET, tetracycline; TGC, tigecycline; CST, colistin.
